# Multiple Sclerosis Gene Therapy Using Recombinant Viral Vectors: Overexpression of IL-4, IL-10 and Leukemia Inhibitory Factor in Wharton’s Jelly Stem Cells in The EAE Mice Model 

**DOI:** 10.22074/cellj.2017.4497

**Published:** 2017-08-19

**Authors:** Ahmad Hosseini, Hajar Estiri, Haleh Akhavan Niaki, Akram Alizadeh, Baharak Abdolhossein Zadeh, Sayyed Mohammad Hossein Ghaderian, Akbar Farjadfar, Ali Fallah

**Affiliations:** 1 Cellular and Molecular Biology Research Center, Shahid Beheshti University of Medical Sciences, Tehran, Iran; 2 Department of Cell Biology and Anatomical Science, Faculty of Medicine, Shahid Beheshti University of Medical Sciences, Tehran, Iran; 3 Department of Molecular Medicine, School of Advanced Technologies in Medicine, Tehran University of Medical Sciences, Tehran, Iran; 4 Iranian Institute of Cell and Gene Therapy, Tehran, Iran; 5 Cellular and Molecular Biology Research Center, Babol University of Medical Sciences, Babol, Iran; 6 Department of Genetics, Faculty of Medicine, Babol University of Medical Sciences, Babol, Iran; 7 Cellular and Molecular Research Center, Shahrekord University of Medical Sciences, Shahrekord, Iran; 8 Urogenital Stem Cell Research, Shahid Beheshti University of Medical Sciences, Tehran, Iran; 9 Department of Biotechnology, Fasa University of Medical Sciences, Fasa, Iran; 10 BioViva USA Inc, Bainbridge Island WA, USA

**Keywords:** Gene Therapy, Multiple Sclerosis, Wharton’s Jelly Stem Cells, Cytokines

## Abstract

**Objective:**

Immunotherapy and gene therapy play important roles in modern medicine. The aim of this study is to evaluate the overexpression of interleukin-4 (IL-4), IL-10 and leukemia inhibitory factor (LIF) in Wharton’s jelly stem cells (WJSCs) in the experimental autoimmune encephalomyelitis (EAE) mice model.

**Materials and Methods:**

In this experimental study, a DNA construction containing *IL- 4, IL-10* and *LIF* was assembled to make a polycistronic vector (as the transfer vector). Transfer and control vectors were co-transfected into Human Embryonic Kidney 293 (HEK-293T) cells with helper plasmids which produced recombinant lentiviral viruses (rLV). WJSCs were transduced with rLV to make recombinant WJSC (rWJSC). *In vitro* protein and mRNA overexpression of *IL-4, LIF,* and *IL-10* were evaluated using quantitative polymerase chain reaction (qPCR), enzyme-linked immunosorbent assay (ELISA) and western blot (WB) analysis. EAE was induced in mice by MOG-CFA and pertussis toxin. EAE mice were injected twice with 2×10^5^ rWJSCs. The *in vivo* level of *IL-4, LIF, IL-10* cytokines and *IL-17* were measured by ELISA. Brain tissues were analyzed histologically for evaluation of EAE lesions.

**Results:**

Isolated WJSCs were performed to characterize by *in vitro* differentiation and surface markers were analyzed by flow cytometry method. Cloning of a single lentiviral vector with five genes was done successfully. Transfection of transfer and control vectors were processed based on CaPO_4_ method with >90% efficiency. Recombinant viruses were produced and results of titration showed 2-3×10^7^ infection-unit/ml. WJSCs were transduced using recombinant viruses. *IL-4, IL-10* and LIF overexpression were confirmed by ELISA, WB and qPCR. The EAE mice treated with rWJSC showed reduction of *Il-17*, and brain lesions as well as brain cellular infiltration, *in vivo*. Weights and physical activity were improved in gene-treated group.

**Conclusion:**

These results showed that gene therapy using anti-inflammatory cytokines can be a promising approach against multiple sclerosis (MS). In addition, considering the immunomodulatory potential of WJSCs, an approach using a combination of WJSCs and gene therapy will enhance the treatment efficacy.

## Introduction

Autoimmune disease (AD) is defined as a malfunction of human immune system in which reactive immune system cells attack functional cells that will be destroyed following apoptosis (1). When the number of these dead cells reaches a critical number in a specific tissue, physiological functions will be disrupted and pathological symptoms of an AD will appear. Many tissues can be targeted by reactive immune cells (2). So far, over 150 ADs have been identified. Moreover, chronic inflammation is the initiating step in many diseases. Some of the most important ADs are multiple sclerosis (MS), type 1 diabetes (T1D), psoriasis, rheumatoid arthritis (RA), systemic lupus erythematosus (SLE), autoimmune thyroid diseases and inflammatory bowel diseases. Many specialists agree that chronic inflammation leads to ADs (3). Considering the inflammation, cytokines are divided into pro-inflammatory and anti-inflammatory cytokines. Cytokines such as interleukin-4 (IL-4), IL-10, IL-13, leukemia inhibitory factor (LIF), transforming growth factor- beta (TGF-β) and FasL are anti-inflammatory cytokines. 

MS is the most common chronic inflammatory disease in which demyelination occurs in the central nervous system, leading to neurological disabilities. The most common class of MS is relapsing-remitting MS (RRMS) which is present in more than 80 percent of the patients. MS typically starts at the age of 20-30 years, with a female predominance that recently has reached a ratio of 3:1 in comparison to males. Nowadays, there is no treatment to stop the progression of MS or to reverse its neuropathology (4). Anti- inflammatory molecules can be considered as the forefront of MS and other autoimmune diseases. New advanced technologies like gene therapy and immunotherapy, are able to introduce new treatments against MS. Monoclonal antibodies as an effective and targeted immunotherapy, play an important role in modern medicine. Natalizumab, alemtuzumab, rituximab, ofatumumab, and secukinumab are monoclonal antibodies approved for MS treatment. Most of these monoclonal antibodies like secukinumab, inhibit IL-17 or other pro-inflammatory proteins that play a major role in MS initiation and progression (5). 

Moreover, anti-inflammatory gene therapy is another approach against MS. There have been many controversial arguments on the pro- inflammatory and anti-inflammatory functions of human cytokines. In the literature, IL-4, LIF, IL-10 are mentioned as anti-inflammatory cytokines while IL-17 has been accepted as a pro-inflammatory cytokine. Inhibition of pro-inflammatory cytokines and overexpression of anti-inflammatory cytokines can be effective approaches in the gene therapy of ADs (6). IL-4 is a key effector in the differentiation of CD4^+^ cells in to type 2 helper (TH2)cells.IL-4 works as an anti-inflammatory cytokine, TH1 cell suppressor, and intracellular pathogen protector. Tissue macrophages produce IL-4, that in turn suppresses tumor necrosis factor-alpha (TNF-α) and IL-1β. In inflamed tissue, massive production of IL-4, IL-10, and other anti-inflammatory factors such as LIF and IL-27, make TH2 the predominant subtype of T cell. In inflamed tissues, IL-4 activates lipopolysaccharide (LPS) and suppressor of cytokine signalling-1 (SOCS1) as the downstream effectors (7). It has been shown that injection of recombinant adeno-associated virus (AAV) and naked plasmids coding *IL-4* can treat AD in the animal models (8). 

IL-10 is a potent anti-inflammatory cytokine produced by monocytes and lymphocytes. IL- 10 suppresses the expression of many common inflammatory cytokines. Furthermore, IL-10 knockout animals are susceptible to human immunodeficiency virus 1 (HIV-1) infection and rheumatoid arthritis disease (9). IL-10 administration as a naked plasmid, liposomal particle, recombinant adenovirus, naked plasmid and transduced cells, shows therapeutic effects on autoimmune diseases (8). LIF cytokine has protective properties for neuron and oligodendrocyte that makes it a therapeutic candidate for MS. LIF is a pro-inflammatory cytokine with strong immunomodulatory effects as it inhibits TH17 differentiation which enhances neuron myelination by oligodendrocytes. LIF downregulates the autoimmune response by enhancing Treg cell numbers, making it a novel promising treatment for MS and other autoimmune diseases (10). 

Human Wharton’s jelly stem cells (WJSCs) are assembled in large scale from neonatal tissues. WJSCs are pluripotent stem cells with the potential of differentiation into mesodermal, ectodermal, and endodermal lineages (11). These cells possess immunosuppressive activities with minimum stimulation of immune and inflammatory systems, suggesting them as a good cell resource for cell therapy and regenerative medicine. The umbilical cord is a more accessible and minimally invasive source of WJSCs. Umbilical cord WJSCs have a higher proliferation rate in comparison to adult and fetal stem cells (12). However, most of the procedures used for *ex vivo* WJSCs isolation, expansion and differentiation are based on animal or human serum-containing medium, representing a major limitation for clinical applications. 

Immunotherapy and gene therapy play important roles in modern medicine. Here, three anti-inflammatory genes (IL-4, LIF, and IL- 10) were combined in a single lentiviral vector. Overexpression of these genes in WJSCs, which has immunomodulatory properties, might result in an effective co-application of cell and gene therapy for the treatment of the experimental autoimmune encephalomyelitis (EAE) mice model. 

## Materials and Methods

### Polycistronic lentiviral vector construction

In this experimental study, premade dual- promoter lentivector, pCDH-513B was purchased (SystemBio, USA) as a backbone vector. The pCDH-513B contains two promoters namely, cytomegalovirus (CMV) and phosphorus glycerol kinase (PGK). After CMV, multiple cloning site (MCS) is used for gene cloning. PGK promoter mediates the co-expression of *CopA-GFP* (cGFP) and *puromycin* as single mRNA. Cloning of Thosea asigna virus 2A (T2A) self-cleavage peptide between these two proteins sequence leads to separate release of the proteins from the ribosome. The vector is a third generation lentiviral vector with the chimeric Rous sarcoma virus-long terminal repeat (RSV-5ˊLTR) promoter that leads to Tat-independent, 5ˊLTR-GOI-3ˊLTR RNAs transcription in packaging process. 

According to the manufacturer’s protocol, tricistronic human genes of *IL-4, LIF, IL-10* were constructed using Gibson Assembly kit (NEB,USA). Briefly, genes cDNA were purchased (GE Healthcare,USA) and primers were designed with 20 bp overlaps for genes and vector by using online NEBuilder software. Primers were used for amplified genes by using proofreading DNA polymerase, Pfu (Thermofisher, USA). Two P2A self-cleavage peptides were assembled between the genes open reading frames (ORF) to guarantee the monomeric protein release from the ribosome in the translation process. In a single tube, all five DNA fragments were cloned in pCDH-513B, and linearlized with BstBI restriction enzyme (NEB, USA). A few right clones were purified using miniprep plasmid kit (Thermofisher, USA) after transformation and colony PCR. Following confirmation of a few clones by digestion, two correctly digested clones were sent for sequencing. Correct sequence of transfer vector (pCHD-CMV- ILI-EF-GP), that contained *IL-4, LIF, IL-10,* GFP and *puromycin* genes were confirmed by the sequencing. Empty vector which only contained GFP and *puromycin*, was used as the control vector. 

### Human Wharton’s jelly stem cell isolation, expansion, and characterization

Human healthy umbilical cord were obtained from hospitalized children after obtaining the bioethics commission’s approval and paternal agreements. Umbilical cord was washed three times with phosphate-buffered saline (PBS) for removing blood cells and coagulations. Umbili- cal cord amnion membrane was removed and exposed to jelly tissue. After cutting umbilical cord vertically with 2.5 mm surgical punch, one vein, and two arteries were physically removed. About 10-20 umbilical cord pieces were isolated and washed with PBS contained penicillin (200 U/ml) streptomycin (200 µg/ml, Gibco, USA) and amphotericin B (3 µg/mL, Gibco, USA). Umbilical cord pieces were cultured in a six- well plate with a minimum of Dulbecco’s Mod- ified Eagle Medium containing Nutrient Mix- ture F-12 (DMEM/F12) medium (Gibco, USA), supplemented with 30-40% fetal bovine serum (FBS, Gibco, USA). Umbilical cord pieces cul- tured in six-well plates were incubated at 37°C with 5% CO ^2^. The same amount of the medium was added in the next 24 and 72 hours to em- bedded pieces. In the first days of culture, it is critical that tissue sections adhere to plate sur- face to allow cell migration and deviation. After one week, solid umbilical cord pieces were re- moved and cell migration was evaluated under the invert light microscope. The tissues were removed after one week and supplemented with fresh medium until cells reached >70-80% con- fluence. Cells were passaged until they reached the confluency of 80%. 

For adipose differentiation 1×10^5^ WJSCs were cultured in six-well plates. After reaching a 40-50% confluency, an adipogenic differentiation medium (Gibco, USA) was added to the basic medium. Fresh adipogenic medium was replaced every 72 hours for 20-25 days. After about 20 days, cells were fixed with paraformaldehyde, and washed with sterile water and 60% isopropanol. Then, lipid droplets were visualized using Oil Red O (Sigma-Aldrich, USA) staining. In the control group, WJSCs cells were grown in culture medium without adipogenic differentiation medium.

For osteogenic differentiation, a six-well plate was cultured with 1×10^5^ cells per well. After reaching a 40-45% cell confluency, osteogenesis supplement (Gibco, USA) was added to the basic medium. Fresh osteogenic medium was replaced every 72 hours for 20-25 days. After about 20 days, cells were fixed and evaluated using Alizarin Red (Sigma-Aldrich, USA). Alizarin red stains the calcium deposits confirming osteogenic differen- tiation. In the control group, WJSCs were grown in the culture medium without osteogenesis sup- plements. 

### Flow cytometry analysis

WJSCs are positive for specific stem cell markers such as CD44, CD73, CD90 and CD105 but negative for CD34 and CD45 hematopoietic markers. A small fraction of undifferentiated WJSCs, (passage 3, 10^5^ cells) were analyzedusing BD FACSCalibur flow cytometry (BD Bioscience, USA) for the expression of WJSCs surface markers by using specific antibodies at the recommended concentrations. After addition of antibodies, tubes were incubated in the dark at the room temperature for 30-60 minutes. Next, flow cytometry analysis was performed and then data was analyzed using FlowJo (version 7.6.1) software. 

### Recombinant lentivirus production, concentration, and titration

The transfer vector (pCHD-CMV-ILI-EF-GP) and control vector (pCDH-EF-GP) were used for packaging. For the production of recombinant lentivirus (rLV), a 3^rd^ generation system was used. CaPO_4_ protocol was used according to Trono Lab with some modifications. Transfer/ control vector 21 μg , pMD2.G vector 7.5 μg, pMDLg/pRRE vector 15 μg and pRSV-Rev vector 13 μg were dissolved in HEPES buffered water to reach 921 μl. Then, 33 μl Tris-EDTA (TE) buffer was added and the mixture was strongly mixed and left for 3 minutes at room temperature (RT). Next, 105 μl CaCl2 2.5 M was added and the mixture was strongly vortexed. The mixture was left for 3 min for making DNACaCl 2 interaction; then, 1074 μl HEPES 2X was added while the mixture was being vortexed. Finally, 2100 μl master mix was used per 10 cm HEK-293T cells with 70% confluency. Transfection master mix was added as a droplet to all area of the plate. HEK-293T cells medium was changed 2 hours before transfection using 10 ml fresh medium containing 10% FBS.

Transfection medium was replaced with 13 ml fresh medium containing 10% FBS, 14-17 hours post-transfection. After 24 hours, the rate of transfections was determined by counting GFP positive and negative cells under florescent microscope. Supernatant was collected after 24, 48 and 72 hours. Pooled recombinant viruses were passed through 0.24 μm pore filters. Recombinant lentiviral concentration was measured based on polyethylene glycol (PEG) method. PEG 600 50%, NaCl4 M, and PBS were added to pooled recombinant viruses inside polypropylene bottles. Bottles were mixed every 30 minutes and stored at 4°C for 1.5 hours. Then, tubes were centrifuged at 7000 g for 15 minutes at 4°C. According to Trono Lab protocols, recombinant lentiviral titration was proceeded with *WPRE* primers:

F: 5ˊACTGTGTTTGCTGACGCAAC3ˊ 

R: 5ˊCAACACCACGGAATTGTCAG3ˊ and quantitative polymerase chain reaction (PCR) (13). 

Fresh viruses at volumes of 1000, 500, 100, 50, 20 and 0 μl were used for transducing HCT119 cells in a 12-well plate. Concentrated viruses at volumes of 4, 2, 1, 10^-1^, 10^-2^ and 0 μl were used for transducing HCT119 cells in a 12-well plate. Naked transfer vector was used for plotting the standard curve. Recombinant titrated virus were stored at -70°C (with less than three times of freeze-thaw) for future use.

### WJSCs transduction and MTT cell proliferation assay

WJSCs were cultured at a low confluency of 30-40% in a six-well plate. Recombinant viruses from transfer vector and control vector were used in multiple of infections (MOI) 5-10 for transduction. Cell transduction was evaluated using fluorescent microscope, 72 hours after the transduction. Puromycin (1.5 μg/ml) selection started 72 hours after transduction, for the next 5 days. For MTT assay, 8×10^3^ cells were cultured from transduced WJCSs and normal WJCSs in 96-wells plates, 24 hours before the assessment. After 24 hours, MTT reagents were added and incubated for 4 hours. Reaction was terminated with the addition of dimethyl sulfoxide (DMSO) and the plate was read at 570 nm wavelength using BioTek Instruments (Vermont, United States) microplate reader.

### Quantitative polymerase chain reaction

Total RNA was extracted extracted from transduced cells using the mRNA extraction kit (Qiagen, Germany) according to manufacturer’s protocol. Real-time PCR was carried out using 0.5 μg RNA with SYBR Green. Primers used for qPCR are listed in Table 1. Data was presented as the ratio of mean threshold targeted human exogenous genes expression to human endogenous *GAPDH*. For each gene, the specificity of the PCR product was assessed by verifying a single peak on the plots obtained from the melting curve analysis. 

### Western blot analysis

Recombinant WJSCs supernatants were collected 72 hours after transduction. Protein concentrations in the supernatants were determined using a BCA Protein Assay Kit (Thermo Fisher, USA). Then, protein (30 µg/lane) was loaded onto 12% sodium dodecyl sulfate polyacrylamide gel electrophoresis (SDS-PAGE) with protein marker and transferred onto nitrocellulose membranes (Bio-Rad, USA). The membranes were blocked using 5% non-fat milk and immunoblotting was performed using antibodies against IL-4 (Santa Cruz, USA), LIF (Abcam, UK), IL-10 (Santa Cruz, USA) and β-actin (Abcam, UK). Proteins of interest were detected using HRP-conjugated sheep anti-mouse IgG antibody (Abcam, ab6785). Finally, the protein band was visualized by chemiluminescence reagent (ECL) and the integrated optical density (IOD) of each protein band was measured. IOD values were adjusted against the internal standard, β-actin. 

**Table 1:** Human primers used for quantitative PCR for determination of overexpression


Primer name	Primer sequence (5ˊ-3ˊ)	Product size (bp)	Tm

*IL-4*	F: ACTGCACAGCAGTTCCACAG	115	60.10
	R: CTCTGGTTGGCTTCCTTCAC		59.84
*LIF*	F: GTTCCCCAACAACCTGGAC	165	60.21
	R: GGGGTTGAGGATCTTCTGGT		60.31
*IL-10*	F: TTACCTGGAGGAGGTGATGC	147	60.07
	R: GGCCTTGCTCTTGTTTTCAC		59.86
*GAPDHM*	F: CGAGATCCCTCCAAAATCAA	293	60.01
	R: TGTGGTCATGAGTCCTTCCA		60.09


PCR; Polymerase chain reaction and Tm; Melting temperature.

### Experimental autoimmune encephalomyelitis induction and treatment

Female C57BL/6 mice, 7-9 weeks old, were used in this study. EAE was induced according to the standard protocol using MOG35-55 (Sigma-Aldrich, USA) suspended in CFA (Sigma-Aldrich, USA). MOG-CFA emulsion was prepared by adding 200 μg MOG to 100 μl PBS and 100 μl CFA per mouse. MOG-CFA was made for 20 mice after mixing 4 mg MOG with 2 ml PBS and 2 ml CFA. Then, MOGCFA was vortexed for at least 45 minutes within 7-ml Borosilicate glass (Fisher Scientific, USA). After 30 minutes the emulsion was stable. Then, 200 μl MOGCFA per mouse was injected in two dorsal regions of hind limbs and forelimbs. Afterwards, 400 ng of pertussis toxin (PTX, Sigma-Aldrich, USA) was diluted in 200 μl PBS. PTX was intraperitoneally injected for two times: once immediately after the MOG-CFA injection and the other, 48 hours after the MOG-CFA injection. Weights and clinical scores were evaluated daily.

Therapeutic effect of *IL-4, LIF*, and *IL-10* administration was evaluated in three groups of mice with EAE that had been randomly divided. The first group of EAE mice received WJSCs without genetic engineering, the second group of them received WJSCs transduced with control vector and the third group of EAE mice received WJSCs that was transduced with transfer vectors carrying *IL-4, LIF*, and *IL-10* genes. In all groups, cells were collected following trypsinization. Then the cells were suspended and counted before the injection. Each mouse received 2×10^5^ cells through the tail vein two times on days 15 and 20 after EAE induction. 

### *In vitro* and plasma assay of cytokines level

Two months after immunization and 40-45 days after injection of transduced cells, spleen cells were collected, cell suspensions were prepared in 96-well plates, and 50 ng/ml antigen (MOG35- 55 peptide) was added to RPMI medium. After 72 hours of culture, supernatants were collected and analyzed for cytokines using mouse IL-17 ELISA kit (Abcam, UK). Also, for investigation of the level of overexpression, supernatants from transduced rWJSCs were evaluated using enzyme-linked immunosorbent assay (ELISA). After 72 hours of culturing of transduced rWJSCs, supernatants were collected and analyzed for cytokines production using IL-4, IL-10 and LIF ELISA Kits (Abcam, UK). Fifty five days post-immunization, blood samples were collected from the mice to analyze circulating levels of IL-4, IL-10, LIF and IL-17 by ELISA assay according to the manufacturer’s protocol (Abcam, UK). The concentration of each cytokine was calculated based on the plotted standard curve. 

### Tissue processing and histological analysis

Thirty days post-transplantation, the animals were sacrificed. Brain tissues were harvested and fixed in 10% formalin. Fixed tissue was cut in 6-µm sections and stained with hematoxylin and eosin (H&E). Then, brain cellular infiltration, as a physiopathology marker, was evaluated. 

### Weight and clinical scoring

EAE mice were checked for weight loss every 24 hours. Mice were daily monitored for clinical signs of EAE. Typically, EAE is scored on a scale from 0 to 5: A score of 0 shows no obvious changes in motor function in comparison with non-immunized mice; A score of 1 means limp tail; A score of 2 means limp tail and weakness of hind legs; A score of 3 means limp tail and complete paralysis of hind legs or limp tail with paralysis of one front and one hind leg; A score of 4 means limp tail, complete hind leg, and partial front leg paralysis and A score of 5 demonstrates spontaneously rolling in the cage. 

## Results

### Construction of polycistronic lentiviral vector

**Fig.1:**
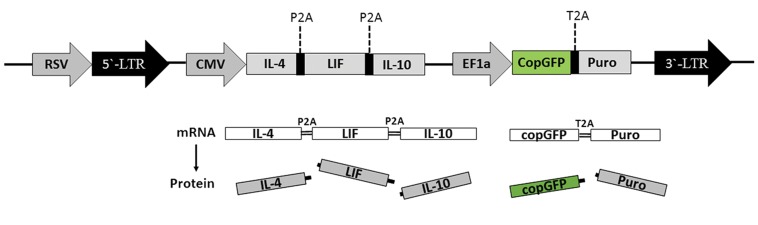
Polycistronic third generation lentiviral vector. Cloning of RSV promoter before 5ˊ-LTR makes Tat-independent and third generation lentiviral vector. RSV makes whole viral genome transcription in packaging process. Two transcriptions derived from CMV and EF1 promoters. Introducing P2A and T2A self-cleavage peptides secures grantee releasing each proteins meanwhile of translation of mRNA in ribosome. This polycistronic lentiviral overexpression five protein simultaneously in transducted cells. RSV; Rous sarcoma virus, LTR; Long terminal repeat, CMV; Cytomegalovirus, EF1; Elongation factor-1, P2A; Porcine teschovirus-1 2A, and T2A; Thoseaasigna virus 2A.

Polycistronic vector containing *IL-4, LIF* and *IL- 10* was constructed using DNA assembling method (14). Sequences of all three genes were confirmed following enzymatic digestion and sequencing. Cloned two P2A self-cleavage peptide sequence after *IL-4* and *LIF* confirmed the release of all three genes which was independent from the ribosome with self-cleavage after glycine and before prolin in P2A protein sequence. In this construct, *IL-4, LIF,* and *IL- 10* mRNA were transcript from CMV promoter and *cGFP* and *Puromycin* mRNA were transcript from EF promoter. Transfer vector expressed five proteins from two transcript mRNAs as shown in Figure 1. 

### Extraction, characterization, and differentiation of WJSCs

**Fig.2:**
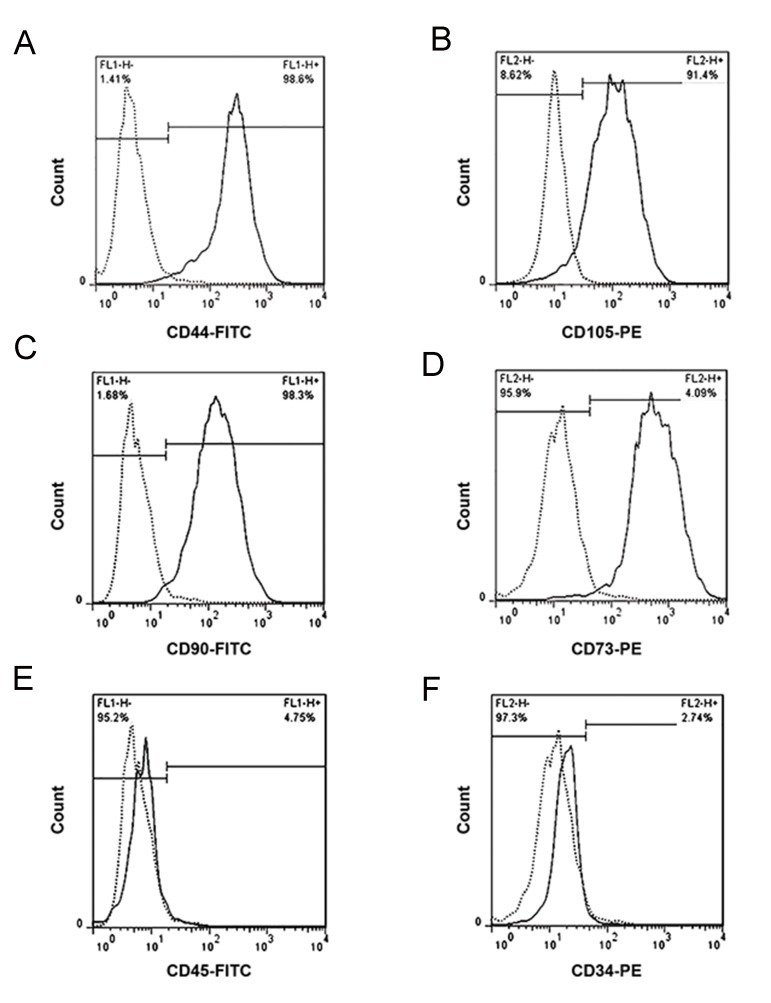
Immunotyping of Wharton’s jelly stem cells (WJSCs). Flow cytometry analysis of WJSCs populations showed positive marker for A. CD44 (98.60%), B. CD105 (91.40 %), C. CD90 (98.30 %), D. CD73 (95.90%) and negative marker for E. CD45 (4.75 %), and F. CD34 (2.74%). Results showed that more than 95% of WJSCs are positive for mesenchymal stem cell markers and negative for hematopoietic stem cells markers.

**Fig.3:**
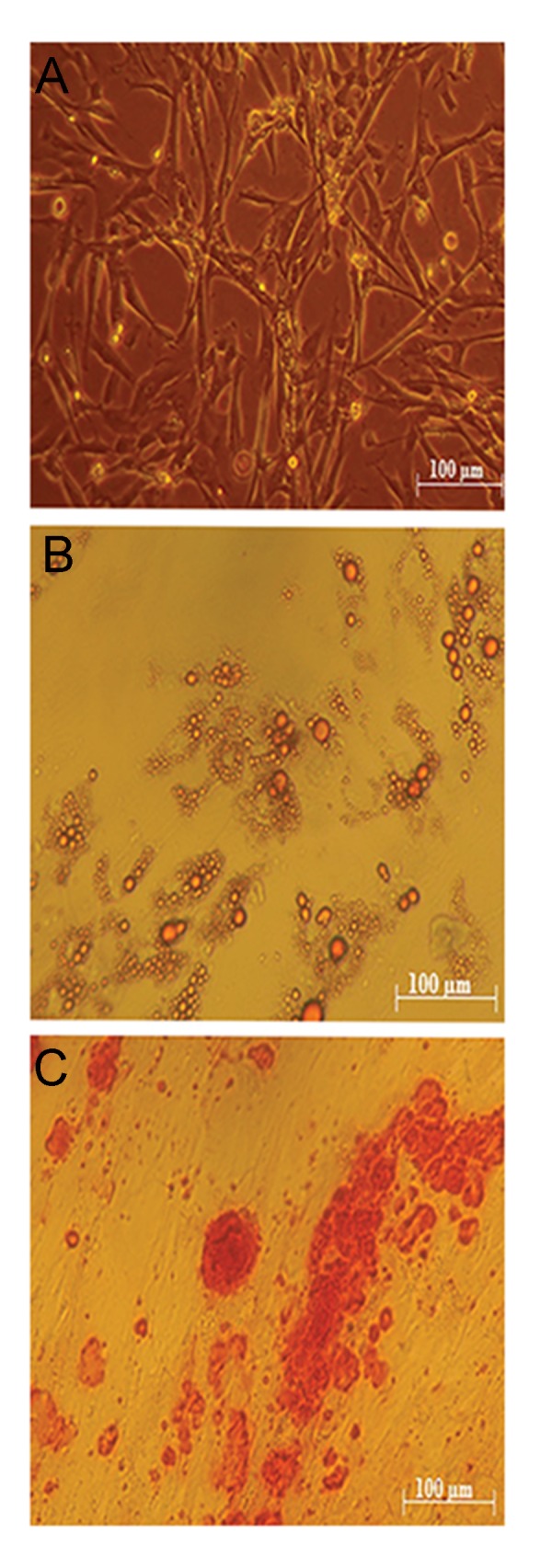
Isolation and diffraction of Wharton’s jelly stem cells (WJSCs) from umbilical cords. A. The isolated WJSCs were cultured in DMEM-F12 and showed fibroblasts morphology, B. As was showed in, oil droplets confirmed adipogenic differentiation, and C. Stained mineral calcium indicated osteogenic differentiation.

The WJSCs were collected from the umbilical cords (n=2) retrieved from healthy full-term women with elective cesarean delivery. Cells were isolated without any enzymatic digestion, only based on cell migration and surface attachment. The vein and artery were removed from umbilical cord sections. Following tissues adhesion and cells migration, fibroblastic-like morphology of the cells were confirmed under invert microscope. Cells were expanded through more than 10 passages with no significant differences among cultures. The success of obtaining human WJSCs without any enzymatic interventions was previously reported (15). Immunophenotyping of cell-surface antigens were done in the third passage and the identity and properties of WJSCs were confirmed by flow cytometry analysis. WJSCs were positive for CD44, CD105, CD90, and CD73 markers and negative for hematopoietic markers, CD45, and CD34 (Fig .2). Also, the capacity of WJSCs in differentiating into adipogenic and osteogenic cells, was evaluated. The accumulation of lipid vacuoles were demonstrated by Oil Red O staining (Fig .3A,B) and calcium deposition was revealed by Alizarin Red (Fig .3C). 

### Recombinant lentiviral production, concentration, titration and transduction 

**Fig.4:**
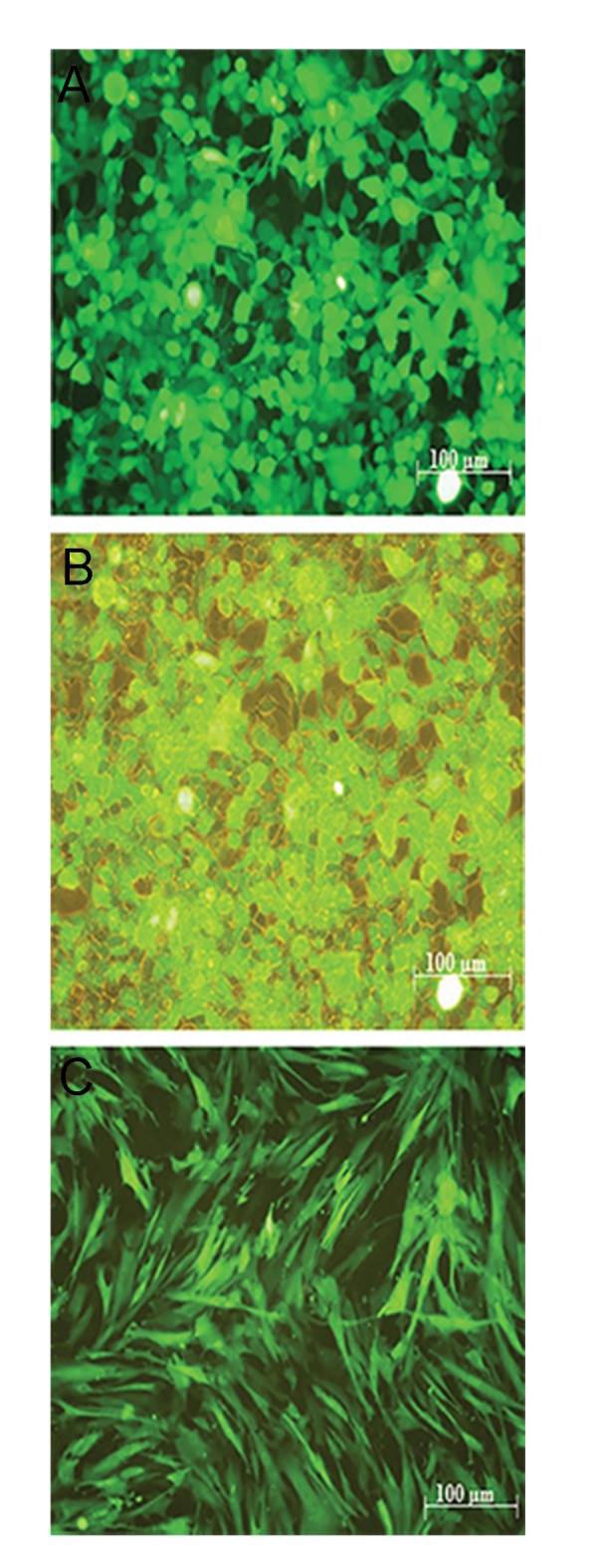
Transfection of lentiviral transfer vector in HEK-293T and transduction of Wharton’s jelly stem cells (WJSCs) were assessed with expression of copaGFP under fluorescent microscope. A. Transfected cells under fluorescent microscope were showed, B. Cells were illustrated simultaneously in open light and fluorescent microscope image. Comparing both pictures shows the high transfection efficiency is higher than 90%, and C. Transduction of WJSCs were verified with expression of copaGFP.

Transfer and control vector were transfected in HEK-293T cells using CaPO_4_ method. Based on GFP positive and negative cells counted under fluorescent microscope, transfection rate was higher than 90% (Fig .4A, B). Recombinant virus titrations were done using quantitative PCR (qPCR), and based on our data, fresh viruses titration was 1-2.3×10^6^ particles/ml. Concentrated viruses titration raised to 2-3×10^7^ infection-unit/ml. Recombinant lentiviral concentration was evaluated using PEG 6000 and the mixture was centrifuged at 7000 g. Results showed that short-term centrifugation of recombinant viruses results in at least 10 times more recombinant viruses. Concentration causes the loss of about 15-20% of initiation recombinant viruses. Cells transduced with fresh viruses without concentration and selection with puromycin demonstrated the desired result. Transduction of WJSCs with concentrated and fresh recombinant viruses (MOI 5-10) does not show any significant difference (Fig .4C). Puromycin (1.5 μg/ml) was used for the selection of transduced WJSCs.

### Cell viability

**Fig.5:**
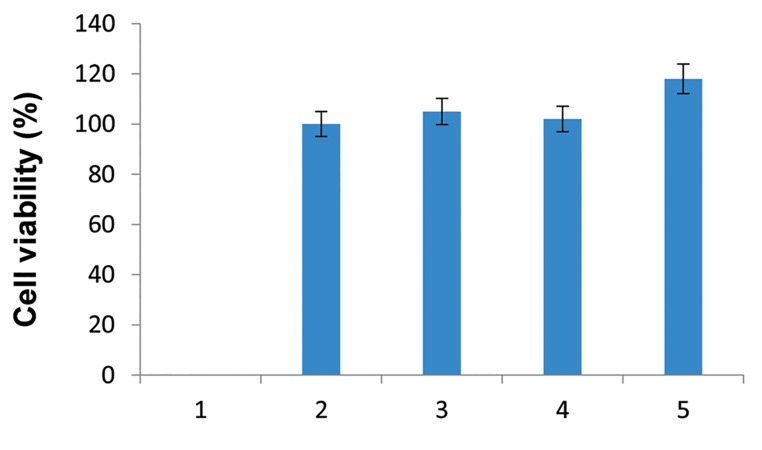
Transducted Wharton’s jelly stem cells (WJSCs) with transfer vector were assayed for viability with MTT test. As showed in picture, cell growth, and viability are the same in transducted WJSCs and normal WJSC cells (P>0.05). Transducted WJSCs viability in different MOIs have no significant versions (P>0.05).

MTT assay showed that transduction and overexpression of genes do not have significant effect on the viability of transcduced cells in comparison with non-transduced cells (P>0.05). These results showed that transduction and genome integration of transfer lentiviral vector did not have any effect on WJSCs viability. Viability in different MOI had no significant differences (P>0.05) as compared with normal WJSCs (Fig .5).

### *In vitro* and *in vivo* gene overexpression assay

*In vitro* expression of *IL-4, LIF* and *IL-10* were confirmed using qPCR and ELISA. QPCR was performed for human *IL-4, LIF,* and *IL-10* exogenous genes against *GAPDH* endogenous gene as control. As shown in Figure 6, exogenous genes were significantly overexpressed in WJSCs transduced with transfer vector in comparison to WJSCs transduced with control vector (P<0.05). For measurement of *IL- 4, IL-10* and *LIF* production by transduced MSCs, an ELISA sandwich was done using supernatants, after 72 hours of culture. The results indicated that *IL-4, IL-10* and *LIF* produced by WJSCs transducted with transfer vector were significantly higher than those of controls (P<0.05).

**Fig.7:**
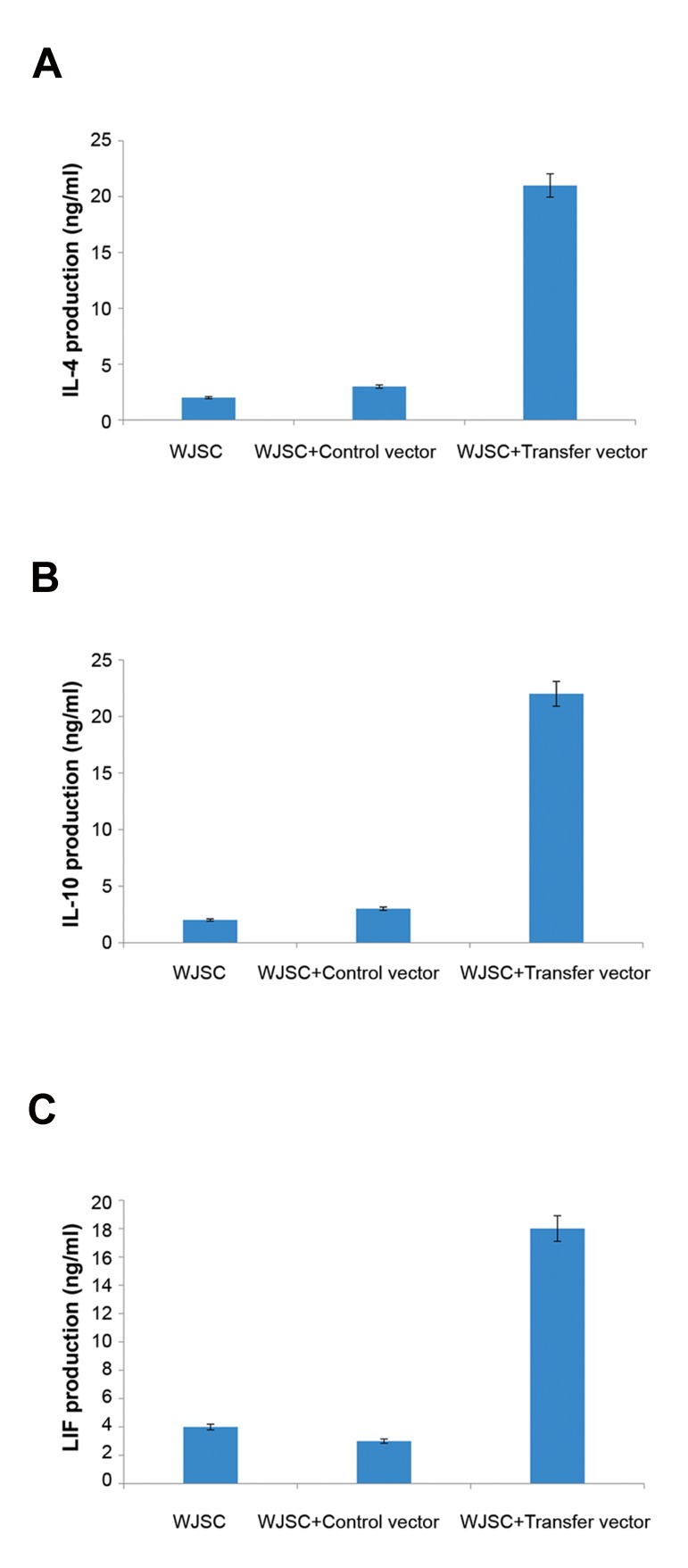
*In vitro* ELISA assay on transducted Wharton’s jelly stem cells (WJSCs) with control and transfer vectors compared here. As shown, there are increase in protein expression level, A. IL- 4, B. IL-10, and C. LIF in transducted WJSCs with control and transfer vectors in compare to other groups. LIF; Leukemia inhibitory factor.

Exogenous human genes of *IL-4, LIF, IL-10* and endogenous mouse *IL-17* were measured using ELISA standard test on plasma obtained from mice that received WJSCs or WJSCs transduced with transfer or WJSCs transduced with control vectors. Bloods were collected from mice tail vein. As shown in Figure 7, expression of antiinflammatory exogenous genes was significantly increased (P<0.05) and *IL-17* pro-inflammatory gene was decreased in treatment group (P<0.05). The Results of *IL-17* ELISA in untreated EAE mice, EAE mice treated with WJSCs, EAE mice treated with WJSCs transduced with control vector and EAE mice treated with WJSCs transduced with transfer vector, indicated that WJSCs has baseline anti-inflammatory properties. Transfer vector with *IL-4, IL-10* and *LIF* decreased *IL-17* level in EAE mice blood.

**Fig.8:**
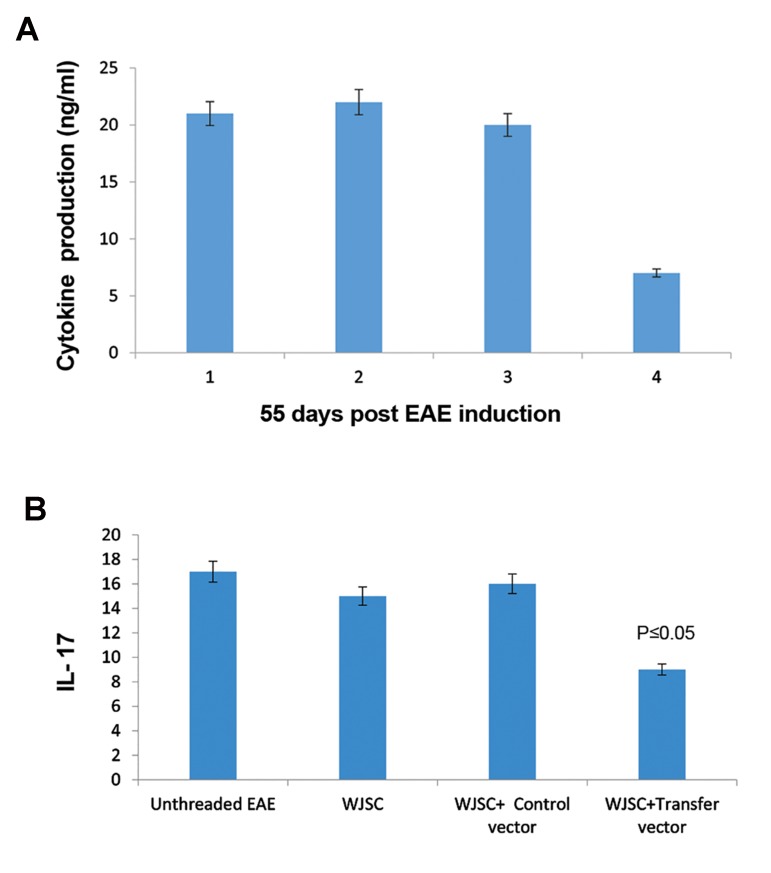
*In vivo* cytosines analysis. A. *In vivo* ELISA assay shows in the group that received Wharton’s jelly stem cells (WJSCs) transducted with transfer vector 55 days post experimental autoimmune encephalomyelitis (EAE) induction. As we see overexpressed genes have therapeutic level about 20 ng/ml in the blood but IL-17 decreased to about 5 ng/ml and B. IL-17 as pro-inflammatory cytokine, indexed EAE induced mice that received different treatment or no treatment.

**Fig.9:**
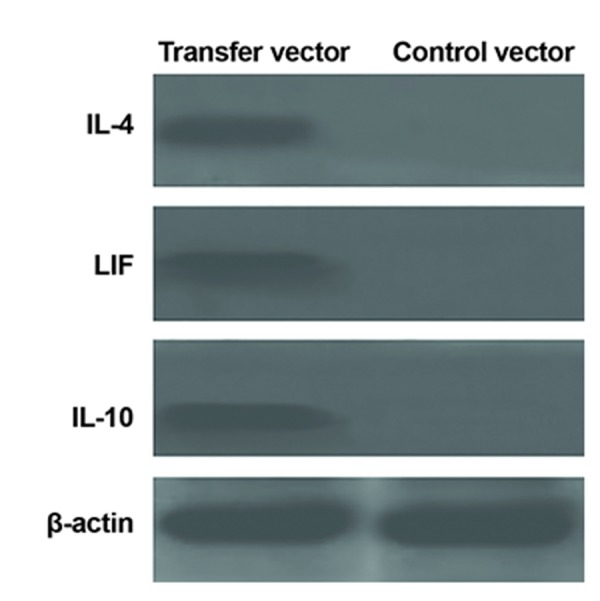
Western blot images here show from transfer vector and control vector after transduction Wharton’s jelly stem cells (WJSCs). As in first column was showed IL-4, IL-10 and LIF expressed from transfer vector. In right column, β-actin only detected as control. LIF; Leukemia inhibitory factor.

Western blot results confirmed gene expression data at the protein level. All three proteins are expressed by transfer vector (Fig .8). Western blot analysis showed similar expression levels for β-actin protein in both control and transduced WJSCs in comparison with IL-4, IL-10 and LIF proteins that were only overexpressed in transduced WJSCs (Fig .9).

These results confirmed the gene transfer and gene overexpression in WJSCs. qPCR showed the overexpression of the genes at the mRNA level. In addition, *in vivo* and *in vitro* ELISA tests confirmed proteins expression in geneticly engineered WJSCs. For more specific confirmation, overexpressed proteins were detected by western blot. These results showed that all proteins were transcribed and translated correctly.

### The EAE mice model and therapeutic gene therapy

**Fig.10:**
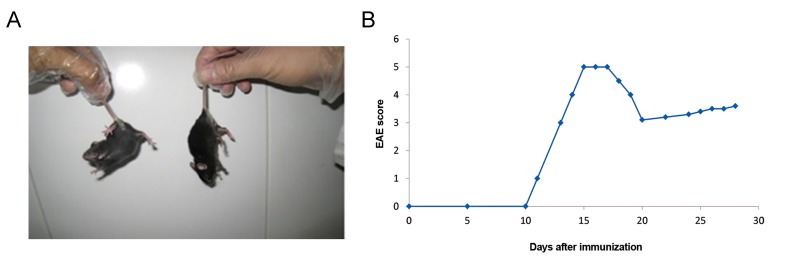
Assessment of experimental autoimmune encephalomyelitis (EAE) induced mice with weight and physical activity measurement. A. Right mouse lost physical activity in compare with left once that can lift the body and B. Image shows scoring of EAE induction.

In our experiment, EAE induction was successful as measuring weights and physical activities of mice confirmed the induction of EAE in mice (with EAE scores of about 4-5, Fig .10).

**Fig.11:**
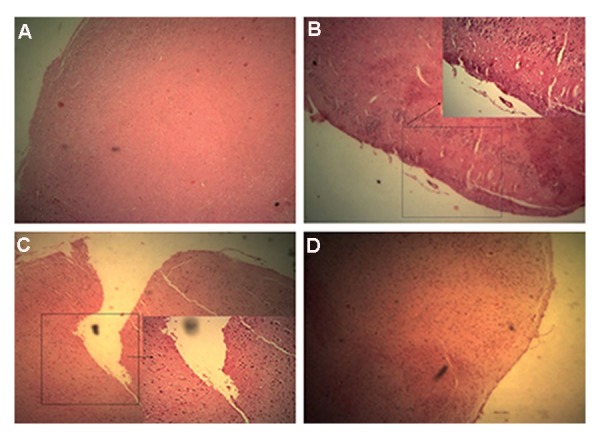
Immunohistology analysis. H&E staining of brain in: A. Normal mice without brain lesions and cell infiltration foci, B. Experimental autoimmune encephalomyelitis (EAE) induced mice with no treatment Fcondensed cell infiltration foci and brain lesion are visible, C. EAE induced mice were treated with WJSCs again cell infiltration and brain lesions are visible but less that untreated group, and D. EAE induced mice treated with WJSC transducted with transfer vector shows more reduction of brain lesions and cell infiltration foci.

*Ex vivo* gene therapy of EAE mice with these cells showed reduction of MS pathology in a mice model. As shown in Figure 11A, in control group without EAE, normal brain tissue was observed. Untreated EAE mice showed brain tissue with wide range of cellular infiltration (Fig ure 11B). Moreover, the result of injection of WJSCs transduced with control virus which only carried cGFP and puromycin are shown in Figure 11C. Immunomodulation and neuroprotection of *IL-4, IL-10* and *LIF* gene therapy in treatment group were confirmed by reductions in IL-17 and brain cellular infiltration (Fig .11D). Weight measurements and physical activity observation indicate EAE recovery after gene therapy. In EAE mice that only received WJSCs without gene therapy, the treatment efficacy was limited but detectable. On the other hand, in EAE mice receiving WJSCs transduced with transfer vector, physical activities and weights were improved.

## Discussion

Autoimmune diseases are the outcome of chronic inflammation. Indeed, many types of autoimmune diseases are due to an imbalance of pro-inflammatory and anti-inflammatory cytokines (16). MS like other ADs has pathologic symptoms of the attacking of immune cells to myelin sheath of the central nervous system neurons (17). A few therapeutic approaches have been proposed for MS teratment. Currently, immunomodulatory and immunosuppressive approaches have reduced the number of relapses but none of them cure the existing deficits nor improve long-term disabilities in MS patients. Small molecules, recombinant proteins and recently, monoclonal antibodies are some of the available therapies for MS (18). Gene therapy and cell therapy combination could be considered as a powerful therapeutic solution for MS treatment in future.

In this study, three pro-inflammatory cytokine genes were constructed as a single lentiviral vector. This DNA was constructed using P2A self-cleavage peptide to guarantee the expression and release of IL-4, IL-10, and LIF. Studies have shown the therapeutic effect of these cytokines in autoimmune diseases (19). In this study, WJSCs were employed as an effective source of stem cells for cell therapy. Pluripotency of these cells was confirmed by flow cytometery and *in vitro* differentiation. In addition, transplantation of these cells showed modest effect on EAE pathology. In the present study, lentiviral was used as a transfer vector for gene therapy. Lentiviral vectors show high efficiency in target cell transduction. Titration of lentiviral vector was done and confirmed that high titers of these recombinant viruses were achieved. Integration of this recombinant virus guarantees the expression of these genes for a long time without reduction of expression as reflected by visualization of cGFP as an index. Random integration of this recombinant virus leads to oncogenicity with integration in prooncogene genes. Despite this limitation, lentiviral vectors are the first choice for gene therapy and chimeric antigen receptors (CARs) T cell therapy in clinical trials (20, 21).

A previous study showed the beneficial effect of *IL-4, IL-10* and *LIF* as autoimmune gene therapy agents (22). In this study, the combination of all these cytokines as a novel autoimmune gene therapy approach was investigated. The ultimate goal of autoimmune gene therapy is to restore and maintain the immune tolerance to the relevant autoantigens and improve the therapeutic effects of cytokines. In this study *IL-4, IL-10* and *LIF* anti-inflammatory gene therapy was combined with immunomodulatory effects of human WJSCs as a novel *ex vivo* gene therapy for ADs. *IL- 17* plays a central role in autoimmune disease. American food and drug administration (FDA) approved monoclonal antibodies against *IL-17* and its receptors, indicating the importance of suppression of this gene and its receptors ADs treatments (22). In this study, it was shown that lentiviral-mediated *ex vivo* overexpression of anti-inflammatory cytokines leads to reduction of *IL-17, in vivo*. Many of autoimmune therapies focus on reduction of *IL-17* and Th17 functions. Here, we demonstrated an efficient method for reduction of *IL-17* in an EAE mice model of MS. The therapeutic advantages of WJSCs and *IL-4, LIF,* and *IL-10* anti-inflammatory cytokines may enable us to develop an effective approach to overcome MS.

This study showed the efficiency of gene therapy in autoimmune diseases. Immunotherapy with recombinant monoclonal antibodies (mAbs) is expensive and most of the poor and undeveloped countries cannot afford recombinant mAbs as a regular therapy. Gene therapy approach for delivery of therapeutic mAbs or proinflammatory cytokines, can be considered as the next generation of immunotherapy. Introducing mAbs genes or therapeutic cytokines allows the patients’ own cells, to express these therapeutic agents without needing recombinant production, purification, and formulation In comparison with short half-life of recombinant proteins in the body, RNA and gene therapy show long lasting protein expression and they are considered as alternatives for recombinant proteins in immunotherapy. *Ex vivo* gene therapy with lentiviral vector could be a promising tool in modern medicine (23). In CAR T cell therapy, as an emerging field of biotherapeutic agents, lentiviral vectors are dominant gene-transfer systems. Many pioneer gene-therapy companies, like Oxford Biomedica and Blubird Bio use lentiviral vectors as gene-transfer systems (24). In addition, combination of lentiviral vectors with novel stem cell sources like WJSCs can make a brilliant perspective for the future of gene therapy usage against autoimmune diseases (25).

## Conclusion

Synergistic expression of three cytokines (IL- 4, LIF, and IL-10) from a single promoter led to more marked anti-inflammatory effects as compared to single gene therapy approaches. In this study, WJSCs which exert immunomodulatory properties, were used as carrier cells for gene therapy. Five genes were overexpressed in WJSCs with high efficiency. Altogether anti-inflammatory genes (*IL-4, LIF,* and *IL-10*), lentiviral vectors and WJSCs combination as and *ex vivo* gene therapy method, could be suggested as a novel gene therapy approach for ADs.
